# A Novel Confocal Scanning Protein–Protein Interaction Assay (PPI-CONA) Reveals Exceptional Selectivity and Specificity of CC0651, a Small Molecule Binding Enhancer of the Weak Interaction between the E2 Ubiquitin-Conjugating Enzyme CDC34A and Ubiquitin

**DOI:** 10.1021/acs.bioconjchem.4c00345

**Published:** 2024-08-21

**Authors:** Joanna Koszela, Nhan T. Pham, Steven Shave, Daniel St-Cyr, Derek F. Ceccarelli, Steven Orlicky, Anne Marinier, Frank Sicheri, Mike Tyers, Manfred Auer

**Affiliations:** † School of Molecular Biosciences, University of Glasgow, Glasgow G12 8QQ, U.K.; ‡ School of Biological Sciences, University of Edinburgh, Edinburgh, Scotland EH9 3BF, U.K.; § College of Medicine and Veterinary Medicine, Institute for Regeneration and Repair, University of Edinburgh, 4-5 Little France Drive, Edinburgh EH16 4UU, U.K.; ∥ Edinburgh Cancer Research, Cancer Research UK Scotland Centre, Institute of Genetics and Cancer, University of Edinburgh, Crewe Road South, Edinburgh EH4 2XR, U.K.; ⊥ X-Chem Inc., Montréal, Québec H4S 1Z9, Canada; # Institute for Research in Immunology and Cancer, University of Montreal, Montreal, Québec H3T 1J4, Canada; ∇ Centre for Systems Biology, Lunenfeld-Tanenbaum Research Institute, Mount Sinai Hospital, Toronto, Ontario M5G 1X5, Canada; ○ Program in Molecular Medicine, The Hospital for Sick Children, Toronto, Ontario M5G 0A4, Canada

## Abstract

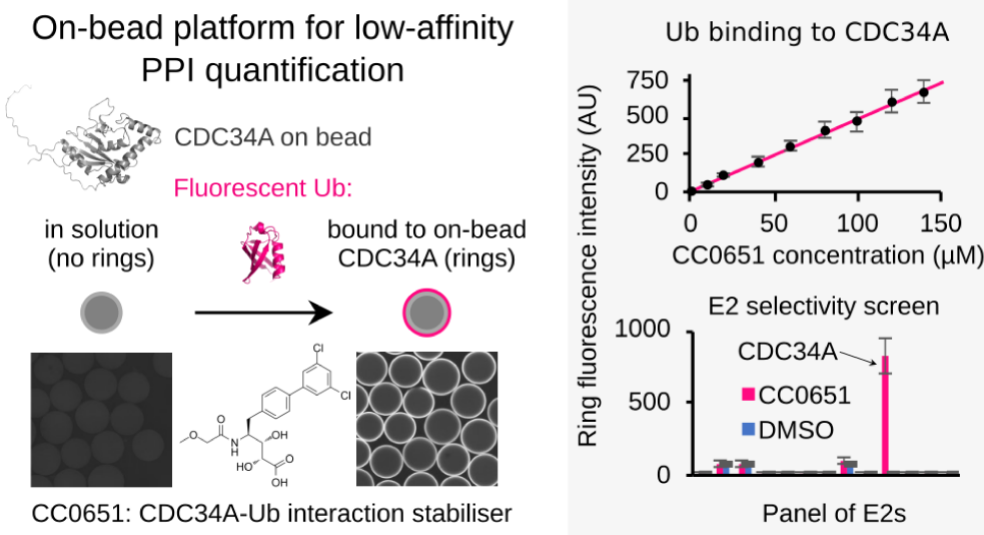

Protein–protein interactions (PPIs) are some of the most challenging target classes in drug discovery. Highly sensitive detection techniques are required for the identification of chemical modulators of PPIs. Here, we introduce PPI confocal nanoscanning (PPI-CONA), a miniaturized, microbead based high-resolution fluorescence imaging assay. We demonstrate the capabilities of PPI-CONA by detecting low affinity ternary complex formation between the human CDC34A ubiquitin-conjugating (E2) enzyme, ubiquitin, and CC0651, a small molecule enhancer of the CDC34A–ubiquitin interaction. We further exemplify PPI-CONA with an E2 enzyme binding study on CC0651 and a CDC34A binding specificity study of a series of CC0651 analogues. Our results indicate that CC0651 is highly selective toward CDC34A. We further demonstrate how PPI-CONA can be applied to screening very low affinity interactions. PPI-CONA holds potential for high-throughput screening for modulators of PPI targets and characterization of their affinity, specificity, and selectivity.

## Introduction

Protein–protein interactions (PPIs) are central to virtually all cellular processes with at least 1 million PPIs experimentally documented in the human interactome to date.^1−4^ Although challenging, PPIs have become attractive drug discovery targets due to their prevalence, diversity, and potential to deliver highly selective modulators.^5,6^ While small molecule inhibitors of PPIs have been discovered and, in a few cases, deployed clinically, the number of such inhibitors remains relatively low.^7−9^ Recently, interaction stabilizers have emerged as an area of intense interest, especially for the bivalent ligand (e.g., PROTAC) and molecular glue classes of molecules that mediate targeted protein degradation through recruitment of targets to E3s for ubiquitination and degradation by the ubiquitin-proteasome system (UPS).^10−12^ Existing methods for discovery of PPI modulators include fluorescence polarization (FP) assays, enzyme-linked immunosorbent assays (ELISA), Förster resonance energy-transfer (FRET) methods, and its variations, microscale thermophoresis and protein microarrays, each of which presents certain limitations and often requires orthogonal assays for hit compound confirmation.^5,13^ Biophysical techniques such as NMR, surface plasmon resonance, isothermal titration calorimetry, and X-ray crystallography are frequently used for hit confirmation and characterization. A major obstacle in screening for PPI modulators is either the extremely high affinity of protein subunits that form stable complexes or, at the other end of the spectrum, the low affinity of often transient interactions that control cellular dynamics. The latter are typically in the high micromolar-to-millimolar dissociation constant (*K*_D_) range and are particularly difficult to detect in assays amenable to high-throughput screens.^14−18^

To address the need for a very sensitive, miniaturized, high-throughput, multiplexed plug-and-play screening technique for identification and characterization of low affinity PPI modulators, we established a microbead-based confocal scanning imaging assay (CONA) called PPI-CONA. CONA was originally developed as an on-bead screening method for one-bead-one-compound (OBOC) chemical libraries.^19,20^ Combining semiautomated single-bead picking and compound labeling for identification and affinity determination of on-bead binders to fluorescently labeled target proteins in solution, OBOC-CONA has yielded a series of new ligands for important protein targets.^21−24^ Bead-based screening has several advantages compared to other fluorescence-based screening techniques, including use of the library synthesis beads as a screening compartment without the need for compound liberation and handling, a small effective assay volume that corresponds to the bead itself, and the availability of linker chemistries that allow facile compound derivatization for secondary validation assays without the need for resynthesis. However, because not all chemical reactions are suitable for solid-phase synthesis, which restricts the breadth of application of OBOC-CONA, we developed CONA techniques that conjugate the target protein, rather than the screening libraries, to different types of beads. CONA has recently proven its versatility in new assays for enzymatic activities within the ubiquitination system,^25^ in mechanistic and screening studies of early-stage alpha-synuclein aggregation,^26^ as well as in detection of RNA-protein interactions^27^ and discovery of protein binding natural products and fluorescent probes.^28^ Encouraged by previous reports around bead-based detection of protein interactions,^29−33^ we reasoned that the on-bead screening system should also be suitable for studying low affinity noncovalent interactions and their modulators, including numerous weak PPIs that dictate enzymatic specificity in the UPS.

Ubiquitin conjugation to substrate proteins is a key post-translational protein modification that controls arguably all cellular processes and, when aberrant, leads to many pathologies. Ubiquitination is mediated by a cascade of E1, E2, and E3 enzymes that catalyze a series of ubiquitin transfer reactions, culminating in modification of a specific substrate protein, either via a single ubiquitin moiety or, more often, ubiquitin chains assembled through various ubiquitin–ubiquitin linkages.^34^ Ubiquitination may target the substrate for degradation by the 26S proteasome or affect target localization, interactions, or activity. The diversity of modifications enabled by different ubiquitin linkages forms a major post-translational signaling code involved in cell cycle, signal transduction, DNA repair, endocytosis, and many other processes. PPIs are key for UPS function, with many enzymatic reactions being initiated via low affinity interactions between UPS enzymes, such as interactions between an E1 activating enzyme and an E2 ubiquitin conjugating enzyme, necessary for ubiquitin transfer.^35,36^ Notably, ubiquitin itself weakly interacts with E2s and other UPS enzymes to properly orient ubiquitin for efficient catalysis and appropriate linkage specificity.^37,38^ Mutational stabilization of specific ubiquitin–enzyme interfaces can block catalysis and enzyme function.^39^ Moreover, these weak interactions can be stabilized by small molecules, as first shown for the compound CC0651 that forms a ternary complex with the human E2 enzyme CDC34A and ubiquitin.^40^ CDC34A, also known as UBE2R1, acts in concert with the cullin-RING E3 enzymes, the largest family of E3 ubiquitin ligases in human cells that recognize a myriad of substrates through a repertoire of hundreds of substrate-specific adaptor subunits.^41^ CDC34A functions redundantly with its close isoform CDC34B (UBE2R2) and other E2 enzymes.^42^ CC0651 specifically stabilizes the normally transient interaction between ubiquitin and the donor binding site on CDC34A, thereby preventing ubiquitin discharge and freezing the catalytic cycle.^40,43^ Recently, a series of more potent CDC34A-ubiquitin interaction stabilizers have been developed through structure–activity relationship (SAR) analysis.^44^

All solution-based assay techniques suffer from low *Z*′-factors if the affinity of the interaction lies in the high micromolar *K*_D_ range. The lower the affinity of a ligand or protein–protein interaction, the lower the *Z*′-value because the assay variability, defined as the standard deviation of high and low controls, increases. In other words, the distribution of high and low controls gets broader which reduces the *Z*′-factor. Besides increasing assay accuracy to achieve more narrow measurement distributions, a second option to improve *Z*′-values is to increase the difference in detection signals between high and low controls. Only nanomolar and low micromolar affinity PPIs will allow for this option, because of the need for a narrow distribution with high signal accuracy. Here, we report PPI-CONA as a new method for detection of low affinity PPIs, exemplified by detection of the weak noncovalent interaction between the CDC34A E2 enzyme and ubiquitin, induced by a small molecule enhancer. In this work, we used PPI-CONA to determine the exquisite selectivity and specificity of this “sandwich” interaction. Importantly, we also extended the method to eliminate false-positive signals. We envisage that PPI-CONA may facilitate the discovery of small molecule modulators of PPIs, as it is suitable for solution-based screening libraries, including but not limited to compounds for PROTAC or molecular glue development and potentially for identification of novel weak protein–protein interactions.

## Results

### PPI-CONA Assay Principle

To develop a bead-based, low affinity PPI-CONA interaction assay, we set up an assay in an analogous manner to UPS-CONA,^25^ whereby a (histidine)6-tagged protein of interest is immobilized at low concentrations at the periphery of Ni^2+^NTA agarose beads prefiltered to a homogeneous size. The on-bead conjugated target protein is incubated in a 384 well-microplate with a fluorescently labeled interacting protein. Upon binding, the interacting protein localizes to the bead periphery and becomes directly detectable by confocal microscopy as a quantifiable “ring” or “halo” originating from fluorescence intensity emission of the dye conjugate. At the low concentrations applied, the ring intensity is linearly proportional to the amount of bound interactors.^20^

### Fluorescent Ubiquitin Binds to Immobilized CDC34A in the Presence of CC0651

We first investigated whether the PPI-CONA assay format is suitable for detection of the CDC34A–Ub interaction. We used this low affinity interaction as an example because of the availability of a small molecule stabilizer, CC0651, which increases Ub binding to CDC34A from millimolar to 14 μM *K*_D_.^43^ CC0651 thus drives complex formation between CDC34A and ubiquitin, as illustrated in Figure S1.

**Figure 1:**
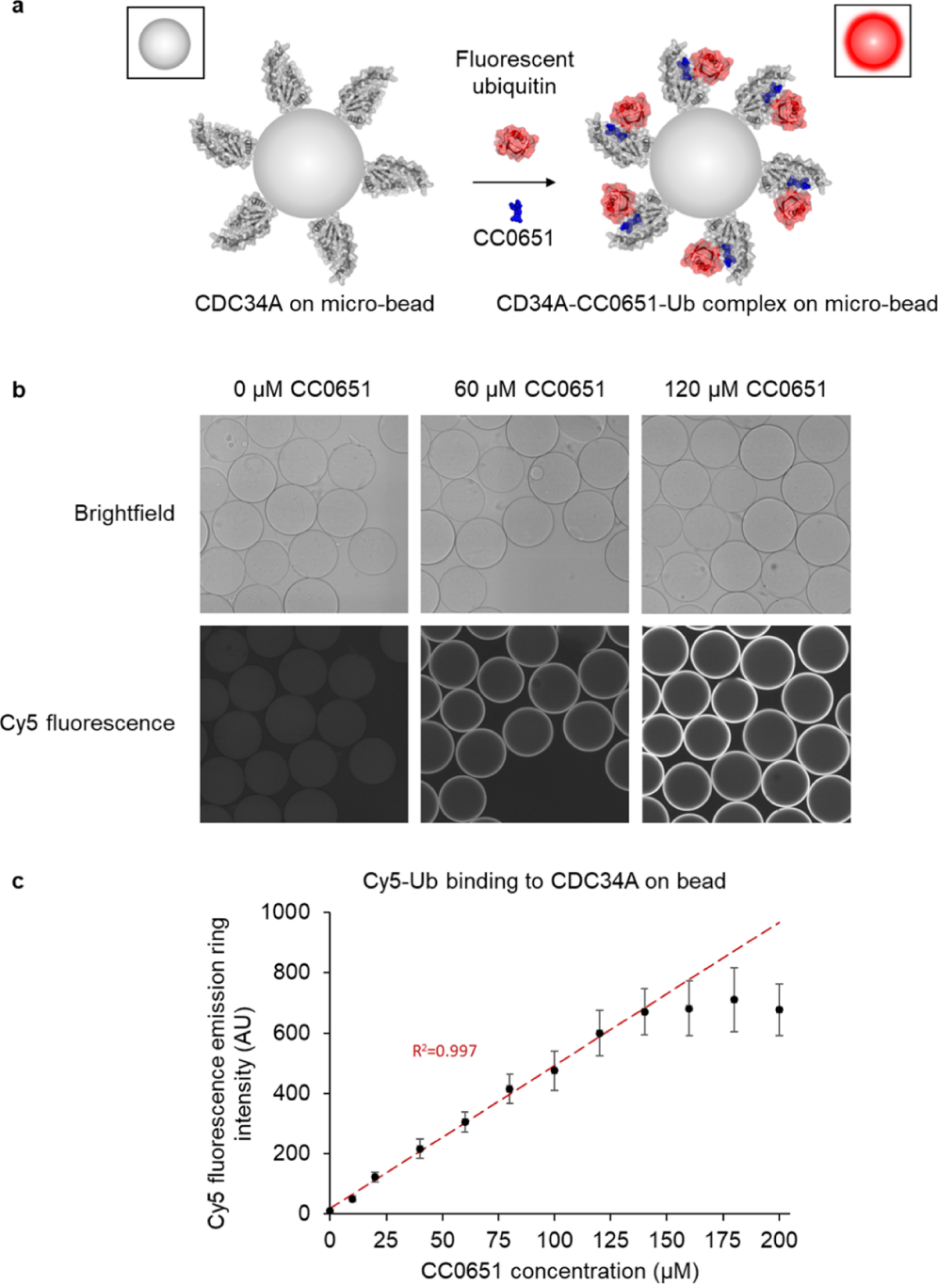
Detection of CDC34A–Ub interaction on bead in the presence of the CC0651 stabilizer. (a) Schematic of the PPI-CONA concept. His6-tagged CDC34A is immobilized on the periphery of Ni^2+^NTA agarose beads and successively incubated with CC0651 and fluorescently labeled Cy5-ubiquitin. Upon ubiquitin binding to on-bead CDC34A, fluorescence emission intensity is confocally detected as a “ring” or “halo” in the confocal imaging plane of the beads. Images based on PDB structure 4MDK.^43^ (b) Example bead images acquired on the Opera instrument (PerkinElmer) in bright-field and Cy5 fluorescence detection channels, with different concentrations of CC0651 as indicated. (c) Cy5 fluorescence emission ring intensities were calculated for increasing concentrations of CC0651. The result of the linear fit to the data points for the concentrations below 130 μM of CC0651 is indicated in red.

CDC34A was immobilized on microbeads and incubated with Cy5-labeled ubiquitin (Cy5-Ub) (Figure 1a). The interaction was monitored by the Cy5 fluorescence emission intensity detected on the bead periphery through confocal microscopy. Under the conditions used and without the small molecular stabilizer present, we were not able to detect the millimolar Ub–CDC34A interaction. However, upon addition of CC0651, we observed an increase in fluorescence emission intensity corresponding to Cy5-Ub binding to on-bead CDC34A, in a compound concentration-dependent manner (Figure 1a–c). Within the tested concentration range, the fluorescence increase was linear up to 130 μM CC0651 (Figure 1c). To ensure that we detected genuine Ub binding and not an artifact due to the fluorophore, we tested Ub labeled with two other dyes: Cy3 and FITC. Consistently, we obtained a fluorescent ring signal only in the presence of CC0651 and not with the DMSO control (Figure S2).

### Apparent *K*_D_ Determination Using PPI-CONA

**Figure 2:**
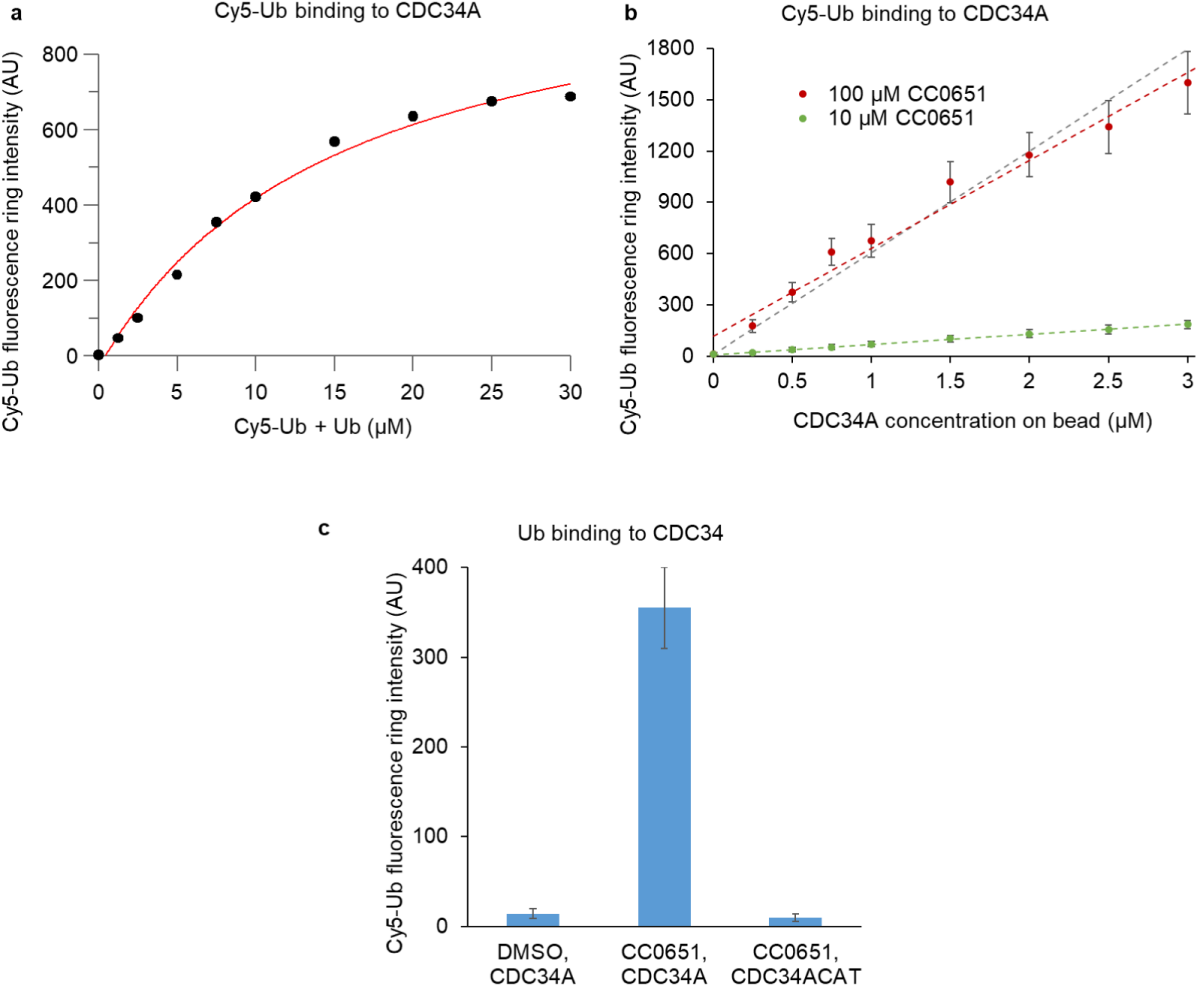
Determination of the apparent on-bead *K*_D_ for CDC34A–Ub interaction. (a) 20 picomoles of CDC34A corresponding to a final concentration of 1 μM in the well was immobilized on bead, incubated with 100 μM CC0651 and increasing concentrations of a 1:40 Cy5:unlabeled ubiquitin mix. The red curve shows a two-parameter (*K*_D_ and *y*_max_) fit to the solution of a quadratic 1:1 binding equation and results in a *K*_D_ of 12.2 ± 2.6 μM. (b) 0.5 to 3 μM concentrations of CDC34A were immobilized on bead and incubated with 10 or 100 μM CC0651 and 250 nM of Cy5-Ub. Linear fits are plotted as dotted lines. (c) CDC34A full length or CDC34A short (“tail-less”) version (CDC34ACAT) was immobilized on bead and incubated with 100 μM CC0651 or with DMSO as a control. Only a full-length CDC34A enzyme showed Ub binding in the presence of CC0651.

We next performed a series of experiments using increasing concentrations of Ub in solution and/or increasing amounts of CDC34A on the bead (Figure 2). To reach saturation in the Ub titration experiment and to avoid fluorescence emission detector saturation on the microscope, we used a 1:40 mix of labeled to unlabeled Ub. The measured average values of Cy5 fluorescence ring intensity corresponding to Cy5-Ub binding to on-bead CDC34A fitted to the solution of the quadratic equation describing complex formation as a function of total Ub ligand and total (free and immobilized) CDC34A protein concentration. This resulted in an apparent *K*_D_ of 12.2 ± 2.6 μM, which agrees closely with a previously reported *K*_D_ of 14 μM^43^ determined by TR-FRET. This indicates that the results from on-bead measurements mirror results obtained in homogeneous solution. In addition, CDC34A-Ub binding was undetectable in our assay in the absence of CC0651 or when a short version of CDC34A lacking the C-terminal tail was linked to beads (CDC34ACAT, Figure 2c). This is again in agreement with the previously reported results from the TR-FRET assay.^43^

For increasing the concentration of CDC34A on bead, we were limited by the amount of protein we could immobilize on the bead periphery without compromising the sharpness of the fluorescence ring. Indeed, with higher protein amounts, we observed that the protein of interest was attached not only on the bead periphery but also further into the bead interior. With CDC34A concentrations between 0.25 and 3 μM, and using 250 nM Cy5-Ub, the PPI-CONA assay remained in the linear detection range with the response directly proportional to the CC0651 concentration up to 100 μM. In consequence, our assay provides a linear signal increase as the saturating conditions of CC0651 were not reached (Figure 2b).

### Detection of Weak E2–Ub Interactions

**Figure 3:**
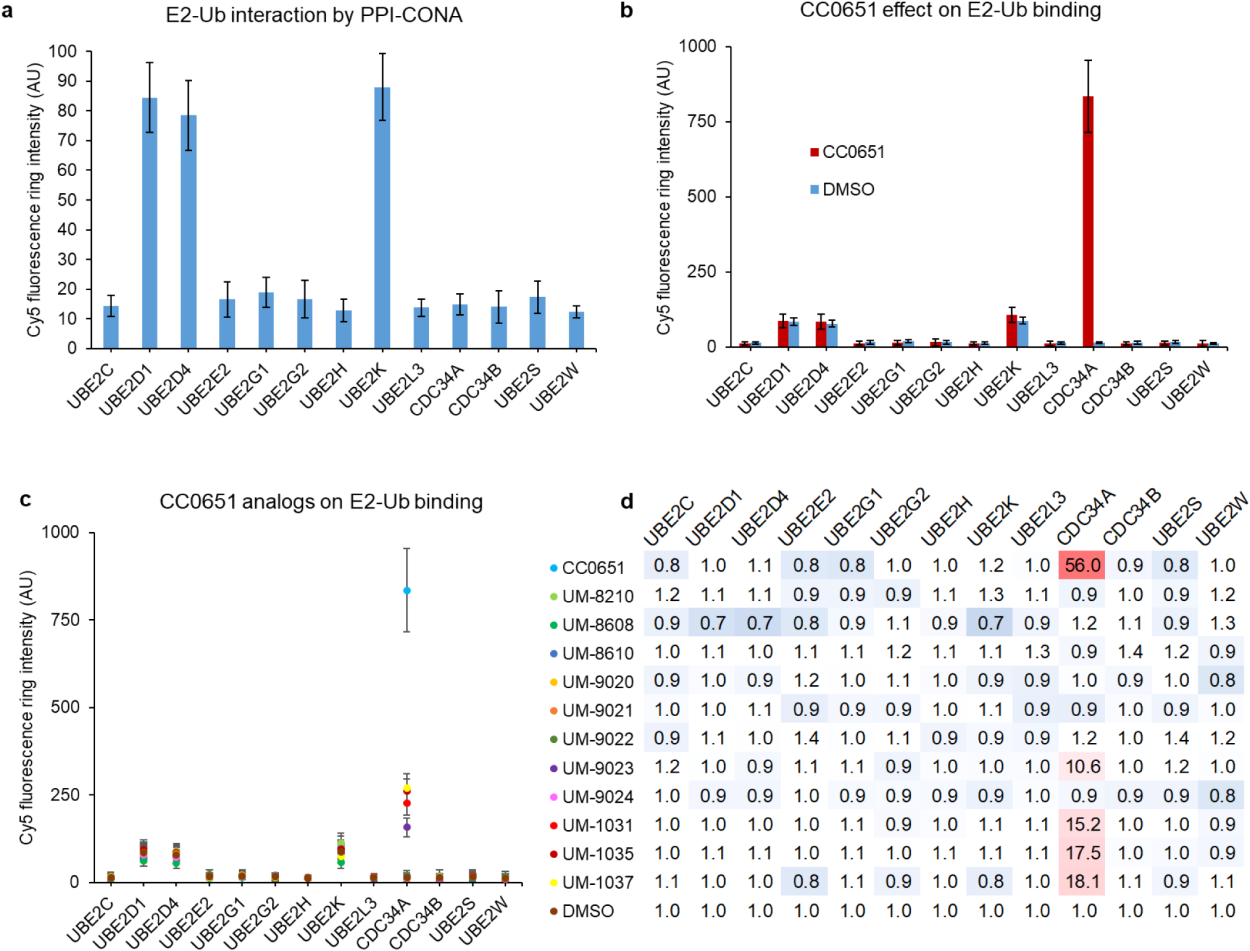
CC0651 is exceptionally selective toward CDC34A–Ub interaction. (a) CDC34A and 12 other E2s were tested in PPI-CONA for Cy5-Ub binding. Twenty picomoles of each His6-tagged E2 was immobilized on 1 μL of 50% Ni^2+^NTA beads prepared as described in Methods, washed, placed in a 384-well plate, and incubated with 250 nM of Cy5-Ub prior to confocal imaging on the Opera reader. For each E2 enzyme, the average ring intensity corresponding to Cy5-Ub binding to E2 is shown. Ub binding was detected for UBE2D1, UBE2D4, and UBE2K. (b) Comparison of E2-Ub binding in the absence (DMSO, blue bars) and presence of 100 μM CC0651 (red bars). E2s were prepared as in (a), with addition of DMSO or CC0651. CC0651 enhanced binding of Ub only to CDC34A. (c) In addition to CC0651, 11 other analogues (colored dots assigned to each compound defined in (d)) at 100 μM were tested as in (b). (d) Specificity binding matrix of Ub to a range of E2s in the presence of CC0651 and the 11 CC0651 derivatives. Shown are ratios of compound-induced increases of Ub binding versus the DMSO control, with red denoting an increase and blue a decrease. CC0651 was the most active among its analogues and selective to CDC34A.

With the assay system established for the CDC34A–Ub interaction, we sought to expand our platform for other E2 enzymes. There are currently 36 ubiquitin-conjugating E2 enzymes described in the human genome. A few E2s are known to engage in noncovalent interactions with ubiquitin, especially in the context of the thioester-bound ubiquitin.^38,45,46^ To test E2–Ub interactions by PPI-CONA, in addition to CDC34A, we prepared another 12 E2s that perform various functions. Under the conditions optimized for CDC34A–Ub interactions as described above, we detected weak direct binding of Cy5-Ub to on-bead UBE2D1, UBE2D4, and UBE2K, but not to any other tested E2 (Figure 3a). Interestingly, binding affinities of UBE2K and the E2s from the UBE2D family to Ub were previously reported. Specifically, NMR titration experiments yielded a *K*_D_ of ∼300 μM for UBE2D3 (UBCH5c)/Ub complex.^47^ A reported *K*_D_ obtained with NMR for UBE2K (E2–25K)/Ub was 1–1.5 mM,^48,49^ while SPR data for the isolated UBA domain of UBE2K binding to Ub indicated a *K*_D_ of ∼400 μM.^50^ Detection of UBE2K–Ub and UBE2D1/D4–Ub interactions by PPI-CONA suggests that the platform is suitable for detection of weak interactions, stabilizers, and inhibitors in the submillimolar *K*_D_ range.

### CC0651 Is Exceptionally Selective toward Stabilizing the CDC34A–Ub Interaction

Application of substructure searches and a standard battery of molecular similarity techniques,^51^ including USRCAT,^52^ FP4, and ECFP4,^53^ revealed no commercially available CC0651 derivatives or close similars. We note that an irreversible inhibitor based on CC0651 was designed and synthesized for UBE2G2.^54^ Recently, a new series of CDC34A inhibitors was developed through structure-guided design informed by the CC0651 binding pocket in the CDC34A–ubiquitin complex, resulting in discovery of a compound with greatly enhanced potency as compared to CC0651.^44^

Here, we tested CC0651 and a series of analogues on CDC34A and other E2s to verify the compound binding specificity and target selectivity. So far, no comprehensive CC0651 binding studies have been reported since published experiments have been limited to the human E2s CDC34B, UBE2D2, UBE2L3, UBC13/UEV1a, and the yeast CDC34, for all of which no effect was detected.^43^ A weak interaction stabilization effect was however observed on the activity of the trypanosomal homologue of CDC34A.^55^ For a more comprehensive selectivity study, we tested CDC34A and 12 additional human E2s in a PPI-CONA Cy5-Ub binding assay in the presence of CC0651. No effect—either positive or negative—was observed on any other tested E2 under any conditions tried (Figure 3b), indicating the high selectivity of CC0651 toward CDC34A. To confirm that the detected binding differences were not due to nonhomogeneous protein loading on bead, we repeated the experiment with a set of emerald green fluorescent protein (emGFP)-fused E2s, where the emGFP fluorescence signal corresponding to the protein amount was used for normalization. While Cy5-Ub seemed to bind with a lower affinity to emGFP-fused CDC34A, compared to CDC34A, again no binding to other E2s was detected (Figure S4). We then investigated the effects of the 11 CC0651 analogues,^43^ which were available to us and not subject to IP restrictions, on a range of E2s. Compound UM0129023 and three carboxy analogues of CC0651 (UM0131031, UM0131035, and UM0131037) showed a moderate enhancement of the CDC34A–Ub interaction; however, no significant effect of any of the analogues on other E2s was observed (Figure 3c,d). Importantly, we observed a correlation between our fluorescent readout, that is, the Cy5-Ub fluorescence emission ring intensity, and the EC_50_ values of CC0651 and its analogues, previously reported in TR-FRET binding assays^43^ (Figure S2). However, we also noticed a subtle but consistent decrease in the bead ring fluorescence intensity in all E2s with UM0128608. This prompted us to investigate the bright-field images acquired on the Opera instrument, which revealed that the fluorescence decrease could be due to compound precipitation (Figure S3). Further investigation revealed that the compound UM0128210 also precipitated under assay conditions (Figure S3). The observed precipitation can be explained by a reduced solubility of UM0128608 and UM0128210 due to a benzohydrazide and a hydroxy pyrrolidinone group, respectively.

### Discussion

OBOC on-bead library screening^56−64^ including our own OBOC–CONA technique^19−24^ with compounds synthesized on bead has proven successful over the years as documented in a substantial number of publications on technology developments and identifications of binders to PPI targets. The two issues that remained unsolvable are (a) the limited number of scaffolds suitable for solid-phase synthesis and, more importantly, (b) the complex and inconsistent ligand binding thermodynamics of proteins to small molecules presented on the TentaGel bead matrix. For example, if a basic ligand, for example, arginine-containing peptide, is presented to an acidic target protein, millimolar solution binding *K*_D_s often results in nanomolar on-bead binding *K*_D_ values. If an acidic ligand on bead is presented to a basic target protein (e.g., RNA binding proteins) the surface and solution *K*_D_s are nearly the same. This effect appears to be based on enthalpic rather than entropic characteristics, as understood from a substantial number of OBOC-CONA screens performed in a big pharma company (unpublished data and^20^).

With these issues in mind, we explored presenting the target in various flexible ways on bead to proteins, DNA, RNA, and small molecule libraries, thereby developing a more broadly applicable method of bead-based screening. Indeed, with the rise of interest in targeted protein degraders such as molecular glues and PROTACs,^65^ the field of compound discovery for PPIs would benefit from sensitive and high-throughput screening methods for in-solution chemical libraries. As a further application to the repertoire of “reverse” CONA screening techniques, here we developed a highly sensitive on-bead protein–protein interaction and inhibition assay, based on confocal nanoscanning, which allows the precise detection of very low affinity interactions up to the submillimolar *K*_D_ range. This new technique, termed PPI-CONA, rests on a straightforward experimental design and was applied to study the selectivity and specificity of a small molecule CDC34A–Ub interaction stabilizer. Our results revealed an exceptionally high specificity of the small molecule CC0651 toward the CDC34A–Ub interface^40,43^ and suggest that PPI-CONA may be used for high-throughput screens to identify selective and potent inhibitors of other E2 enzymes.^25,66,67^ In addition, PPI-CONA allowed us to detect native interactions with submillimolar affinities between a set of known ubiquitin-interacting E2s (UBE2D1, UBE2D4, and UBE2K) and ubiquitin, thereby proving the assay capable of detecting low affinity and novel PPIs.

Historically, high-throughput screens for PPI inhibitors have had the lowest hit rate of all prominent target classes.^68,69^ This dearth of hits has, in part, been due to poor assay sensitivity in the identification of binders or inhibitors at the standard 10 μM library compound screening concentration. Our PPI-CONA technique performs with high accuracy against known low affinity interactions and is therefore poised to facilitate the discovery of novel PPI modulators. The tight concordance between interaction affinities estimated with the PPI-CONA method here and previously published values based on in-solution assays^43^ suggests that PPI-CONA accurately mirrors solution interaction affinities. Furthermore, we fully exploited the PPI-CONA setup, namely, the unconventional use of the bright-field channel in screening, absent in other fluorescence-based methods such as FRET, to eliminate false-positive hits coming from compound precipitation or protein aggregation. This is an important point as false positives have been a major challenge for high-throughput PPI screening.^68^ However, as with any other fluorescence-based assays, the effects of the fluorophore conjugate need to be controlled through benchmarking against solution-based assays with unlabeled target proteins. PPI-CONA is amenable to automated bead handling and streamlined image processing for applications in medium- and high-throughput screening campaigns.^70^ Beyond facilitating screening for inhibitors and activators of notoriously difficult PPI targets, we envisage that the PPI-CONA method could also be used for identification of key residues in PPI interfaces through systematic mutational analyses and for discovery and characterization of physiologically relevant PPIs using recombinant proteins or affinity-purified proteins from cell lysates.

## Experimental Procedures

### Compounds and Proteins

CC0651 and analogues were synthesized as described.^43,44^ Structures are presented in Table S1 and synthesis routes of new compounds in Figures S5–S10. The masses and purity of compounds were confirmed by analytical HPLC and LC-MS. FITC-ubiquitin was purchased from Life Technologies (cat. no. PV4378) and Rhodamine Red-Nedd8 from BostonBiochem (cat. no. UL-835).

### Protein Expression and Purification

Cysteine-inserted ubiquitin (Cys0-Ub) was expressed from the pETM-30 plasmid, CDC34ACAT (residues 7–184), UBE2D1 and UBE2D4 were expressed from pProEx Hta, and other E2s were expressed from the pET28a-LIC vector. E2 encoding sequences were cloned into the pRSET vector to obtain emGFP-fusion proteins. Recombinant E2 proteins were expressed and purified using standard His6-tag purification, and ubiquitin was expressed, purified, and labeled with Cy5 or Cy3 maleimide on the inserted unique cysteine residue as described previously.^25^

### Preparation of Protein on Bead Conjugates

Beads were prepared as described.^25^ Briefly, nickel nitrilotriacetic acid (Ni^2+^NTA) agarose microbeads were purchased from Qiagen (cat. no. 30250) and filtered using 100 and 120 μm filters (Corning cat. no. 352360, Millipore cat. no. NY2H04700) to obtain beads of homogeneous diameters. Beads were washed thoroughly with binding buffer (0.3 M NaCl, 20 mM HEPES, pH 7.5, 0.01% Triton X-100) and resuspended to obtain a 50% slurry. One microliter of filtered beads was used for each well of a 384-well plate. The volumes and amounts were scaled up according to the number of wells. The beads were incubated with 20 picomoles (1 μM concentration in a final 20 μL volume) or as indicated of His6-tagged E2 protein in ice-cold binding buffer on a shaker at 1000 rpm for at least 20 min at 4 °C. After incubation, the beads were extensively washed with reaction buffer (100 mM NaCl, 20 mM HEPES, pH 7.4, 5 mM DTT) and the volume of bead solution was adjusted to 10 μL per well.

### On-Bead Detection of Protein–Protein Interactions

Beads with attached proteins were distributed in 10 μL volumes into microplate wells (black, flat glass bottom 384-well plate, MMI PS384B-G175) using a wide bore pipet tip (Rainin RC-250W). Ten microliter of a reaction mix was added, containing 250 nM Cy5-Ub (or as indicated) and 100 μM (or as indicated) CC0651 in DMSO or DMSO alone for a final 5% DMSO concentration in reaction buffer (100 mM NaCl, 20 mM HEPES, pH 7.4, 5 mM DTT) and mixed thoroughly. Images were acquired on Opera High Content Screening System (PerkinElmer) at 30 μm above the well bottom at 20× magnification with an air lense (20x Air LUCPLFLN, NA = 0.45). Bright-field and fluorescent channels for detection of Cy5, FITC, emGFP, Cy3, or Rhodamine Red were used in the following settings: excitation wavelength 640 nm (Cy5), 561 nm (Cy3 and Rhodamine Red), and 488 nm (FITC and emGFP); emission filters: 690/70 nm (Cy5), 585/40 nm (Cy3 and Rhodamine Red), and 520/35 nm (FITC and emGFP). 35 images from the center of the well were taken to visualize ∼100 beads per well, and a well sublayout with 20% image field overlap was applied to allow consequent image stitching using Grid/Collection Stitching Plugin for ImageJ.^71^ The stitched images from each channel, either brightfield or fluorescent, were analyzed for each well in a well plate using a custom MatLab script as described.^25^ Ratiometric analysis for emGFP-E2s was performed by calculating the ratio of Cy5/emGFP ring intensity of the same bead. The mean bead ring intensity of each well in each channel was calculated as the average bead intensity of all of the beads in the well. For each well, the mean bead ring intensity and standard deviation were calculated.

## Supporting Information Available

Additional figures including structural representation of the CDC34A-CC0651-Ub sandwich, control results for ubiquitin with different fluorophores binding to CDC34A, comparison of PPI-CONA to TR-FRET, examples of compound precipitation, results for Cy5-Ub binding to emGFP-fused E2s, and additional experimental details including compound structures, synthesis, and characterization (PDF)
