# Supplementary material for: A Novel Confocal Scanning Protein–Protein Interaction Assay (PPI-CONA) Reveals Exceptional Selectivity and Specificity of CC0651, a Small Molecule Binding Enhancer of the Weak Interaction between the E2 Ubiquitin-Conjugating Enzyme CDC34A and Ubiquitin
Source: Bioconjug Chem. 2024 Aug 21;35(9):1441–9. doi: 10.1021/acs.bioconjchem.4c00345 (PMC11417995; doi:10.1021/acs.bioconjchem.4c00345)
Supplement: Supplementary file 1 — bc4c00345_si_001.pdf [file bc4c00345_si_001.pdf]

Supplementary information for:

A novel confocal scanning protein-protein interaction assay (PPI-CONA) reveals exceptional selectivity and specificity of CC0651, a small molecule binding enhancer of the weak interaction between the E2 ubiquitin-conjugating enzyme CDC34A and ubiquitin

Joanna Koszela*[1], Nhan T. Pham[2,3], Steven Shave[2,4], Daniel St-Cyr[5,6], Derek F. Ceccarelli[7], Steven Orlicky[7], Anne Marinier[6], Frank Sicheri[7], Mike Tyers[8], Manfred Auer*[2,]

[1] School of Molecular Biosciences, University of Glasgow, Glasgow G12 8QQ, United Kingdom

[2] School of Biological Sciences, University of Edinburgh, Edinburgh, Scotland EH9 3BF, United Kingdom

[3] College of Medicine and Veterinary Medicine, Institute for Regeneration and Repair, University of Edinburgh, Edinburgh EH16 4UU, United Kingdom

[4] Edinburgh Cancer Research, Cancer Research UK Scotland Centre, Institute of Genetics and Cancer, University of Edinburgh, Crewe Road South, Edinburgh, EH4 2XR, United Kingdom

[5] X-Chem Inc., Montréal, Québec H4S 1Z9, Canada

[6] Institute for Research in Immunology and Cancer, University of Montreal, Montreal, Québec H3T 1J4, Canada

[7] Centre for Systems Biology, Lunenfeld-Tanenbaum Research Institute, Mount Sinai Hospital, Toronto, Ontario M5G 1X5, Canada

[8] Program in Molecular Medicine, The Hospital for Sick Children, Toronto, Ontario M5G 0A4, Canada

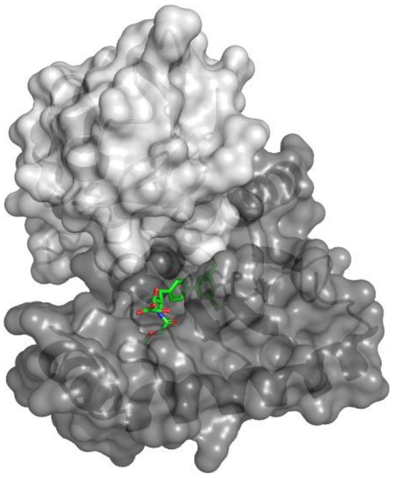

Supplementary figure S1.

CDC34A (dark grey) – Ubiquitin (light grey) – CC0651 (green sticks) ternary complex. Derived from PDBID:4MDK (from PubMed: 24316736)

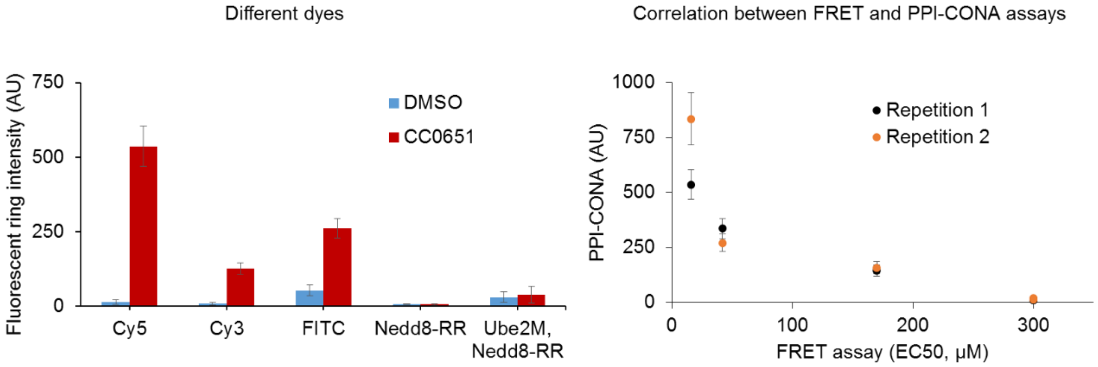

Supplementary figure S2.

Left: Cy5-, Cy3- and FITC-ubiquitin conjugates were tested for interaction with CDC34A in the presence of CC0651 using PPI-CONA. All tested dyes allowed for detection of the interaction, with Cy5 presenting the greatest signal-to noise ratio, as compared to the negative control with DMSO. Right: Comparison of results obtained with two PPI-CONA experiments and the TR-FRET assay as described in [37].

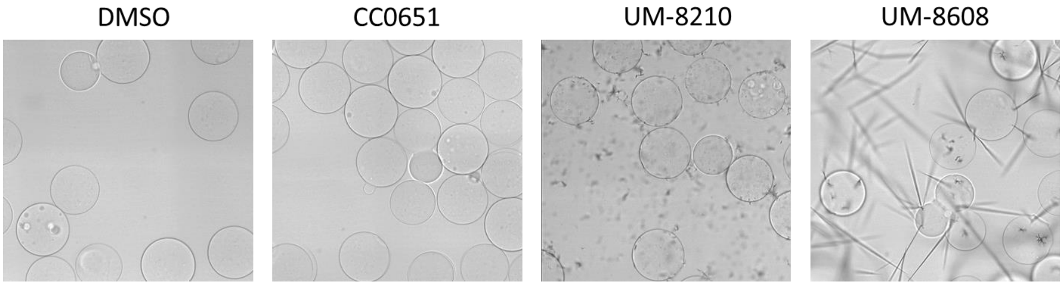

Supplementary figure S3. Examples of compound precipitation observed under brightfield imaging on the Opera™ (PerkinElmer), compared to clean bead view for DMSO and CC0651.

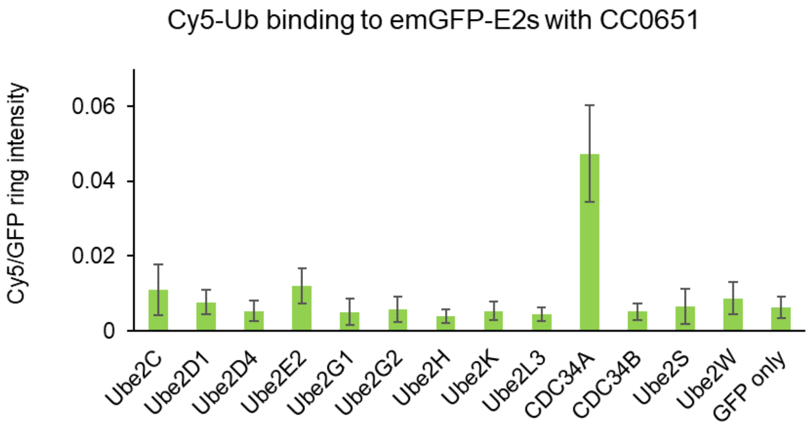

Supplementary figure S4. Cy5-Ub binding to the emGFP-E2s in the presence of CC0651.

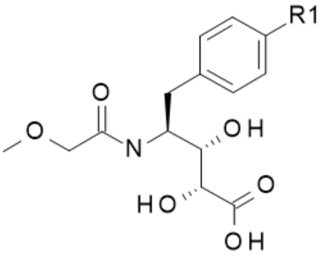		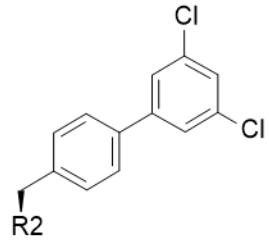	
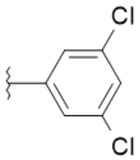	CC0651	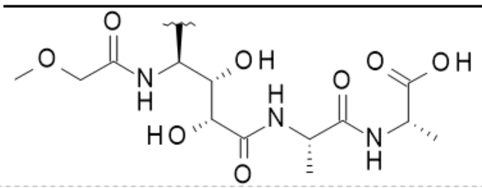	UM0131031
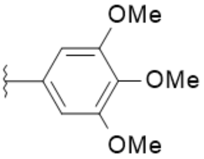	UM0129020	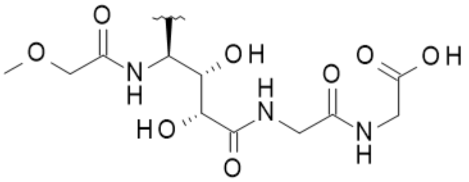	UM0131035
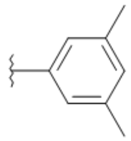	UM0129023	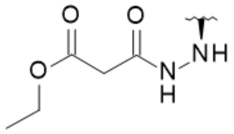	UM0128610
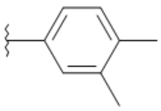	UM0129024	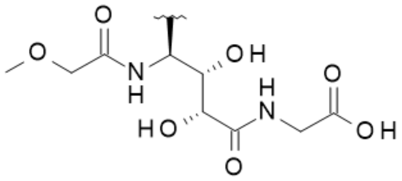	UM0131037
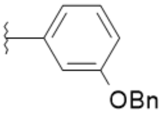	UM0129021	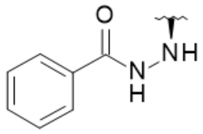	UM0128608
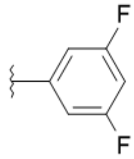	UM0129022	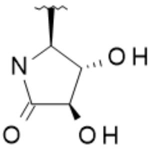	UM0128210

Supplementary Table S1. CC0651 and derivatives assayed with PPI-CONA and evaluated as to their CDC34A-ubiquitin complex enhancing abilities. Actives were determined to be CC0651, UM0129023 and the charged tail modification compounds UM0131031, UM0131035, and UM0131037.

Supporting Methods: new compound synthesis and characterization

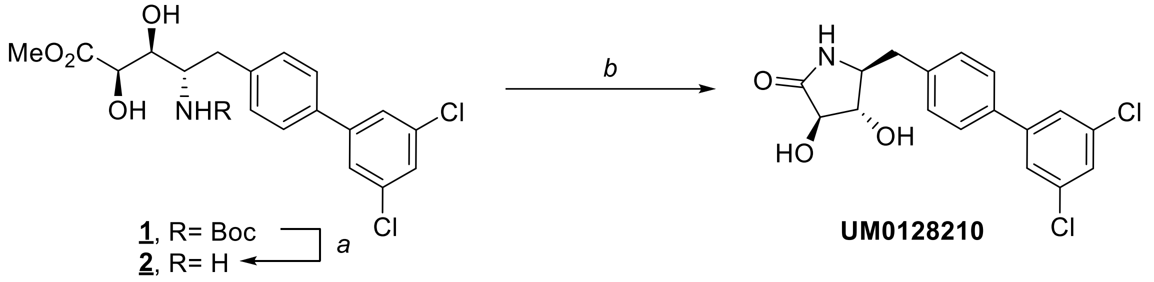

Reagents: a. TFA, CH_2Cl_2, quant.; b. LiOH, THF-H_2O, 90%.

Supplementary Figure 5. UM0128210 synthesis: Boc deprotection of intermediate **1*** (**ref.* H. Huang, D. F. Ceccarelli, S. Orlicky, D. J. St-Cyr, A. Ziemba, P. Garg, S. Plamondon, M. Auer, S. Sidhu, A. Marinier, G. Kleiger, M. Tyers, F. Sicheri, *Nat. Chem. Biol.* **2014**, *10*, 156–63) afforded amino diol **2**, which was saponified to give **UM0128210**.

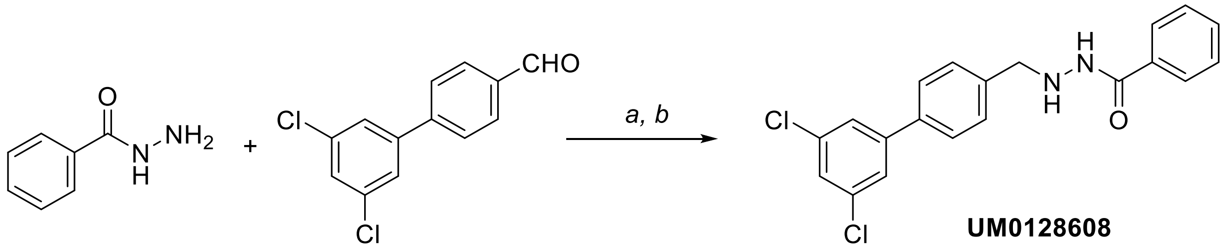

Reagents: a. EtOH, $CDCl_3$; b. Et_3SiH, TFA, 90% overall.

Supplementary Figure 6. UM0128608 synthesis: Reaction of benzohydrazide with 3',5'-dichloro-[1,1'-biphenyl]-4-carbaldehyde gave the intermediate hydrazone, which was submitted to silane reduction to give **UM0128608**.

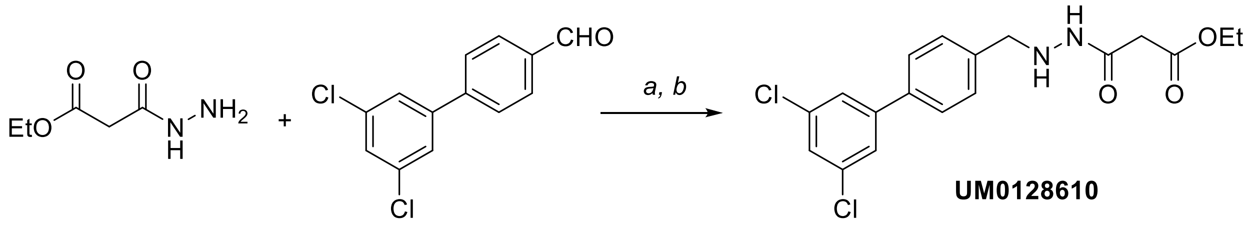

Reagents: a. EtOH, $CDCl_3$; b. Et_3SiH, TFA, 32% overall.

Supplementary Figure 7. UM0128610 synthesis: Reaction of ethyl 3-hydrazinyl-3-oxopropanoate with 3',5'-dichloro-[1,1'-biphenyl]-4-carbaldehyde gave the intermediate hydrazone, which was submitted to silane reduction to give **UM0128610**.

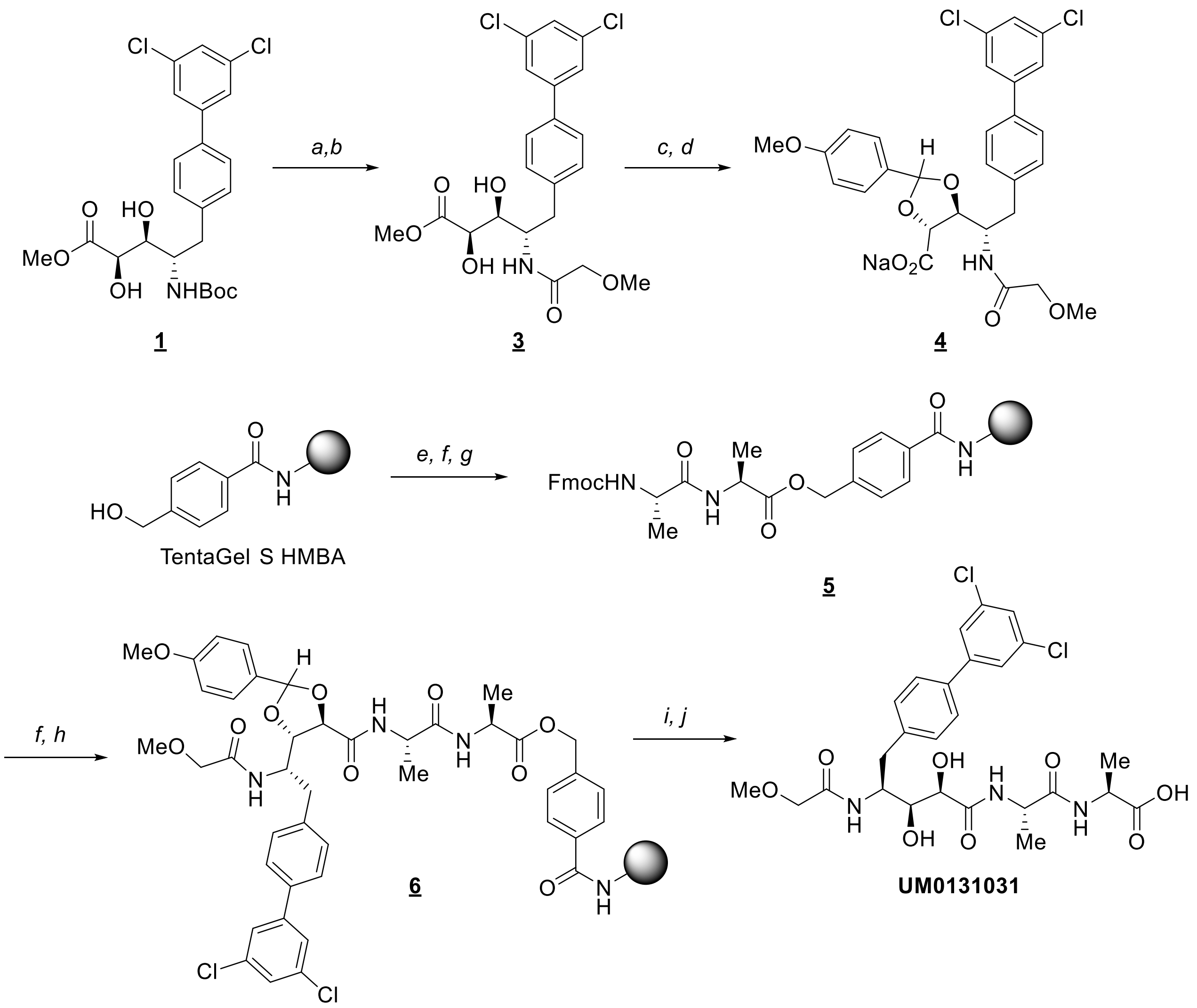

Reagents: a. TFA, CH_2Cl_2, quant.; b. 2-methoxyacetic acid, HATU, TEA, 100%; c. p-anisaldehyde dimethyl acetal, TFA, MeCN, 41%; d. NaOH, dioxane, H_2O; e. Fmoc-Ala-OH, 1-methylimidazole, 1-(mesitylsulfonyl)-3-nitro-1H-1,2,4-triazole, CH_2Cl_2; f. 20% piperidine-DMF; g. Fmoc-Ala-OH, HATU, Hünig's base, DMF; h. **4**, DEPBT, Hünig's base, DMF; i. TFA, MeOH-H_2O; j. LiOH, dioxane-H_2O; AcOH.

Supplementary Figure 8. UM0131031 synthesis: Intermediate **1** (*cf.* **Supplementary Figure 5**) was Boc-deprotected and then submitted to a HATU-mediated amide coupling to give the methoxyacetamide **3**. Acid-catalyzed acetal formation, followed by saponification gave the sodium carboxylate coupling partner **4**. An Fmoc-Ala-Ala capped TentalGel S HMBA resin **5** was prepared using a condensation-deprotection-condensation sequence. Fmoc-deprotection of **5**, followed by amide coupling with **4** gave the acetal **6**. Acid-mediated deprotection of the acetal, followed by base-mediated cleavage of the solid support then afforded **UM0131031**.

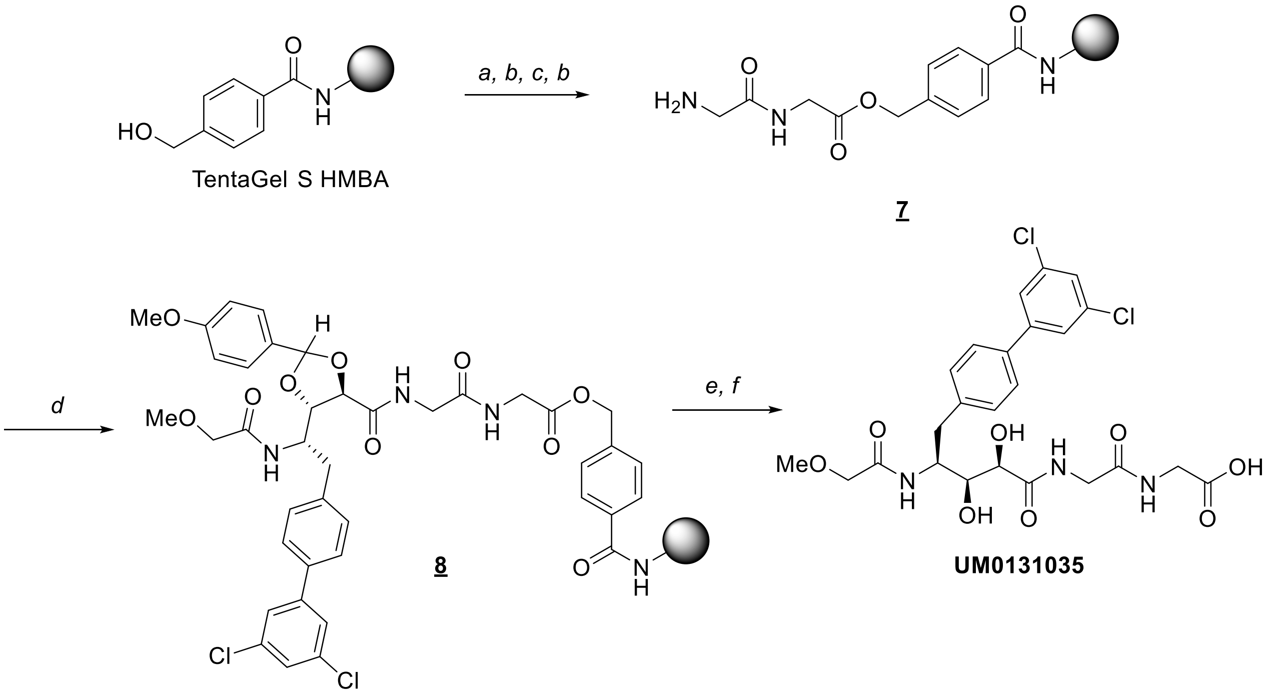

Reagents: a. Fmoc-Gly-OH, 1-methylimidazole, MSNT, CH_2Cl_2; b. 20% piperidine-DMF; c. Fmoc-Gly-OH, HATU, Hünig's base, DMF; d. 4 (acid form), DIC, HOAt, DMF; e. TFA, MeOH-H_2O; f. LiOH, dioxane-H_2O; AcOH.

Supplementary Figure 9. UM0131035 synthesis: An Fmoc-Gly-Gly capped TentalGel S HMBA resin was prepared using a condensation-deprotection-condensation sequence. Subsequent Fmoc-deprotection afforded the amine intermediate **7**. Amide coupling using the acid form of intermediate **4** gave the acetal **8**. Acid-mediated deprotection of the acetal, followed by base-mediated cleavage of the solid support then afforded **UM0131035**.

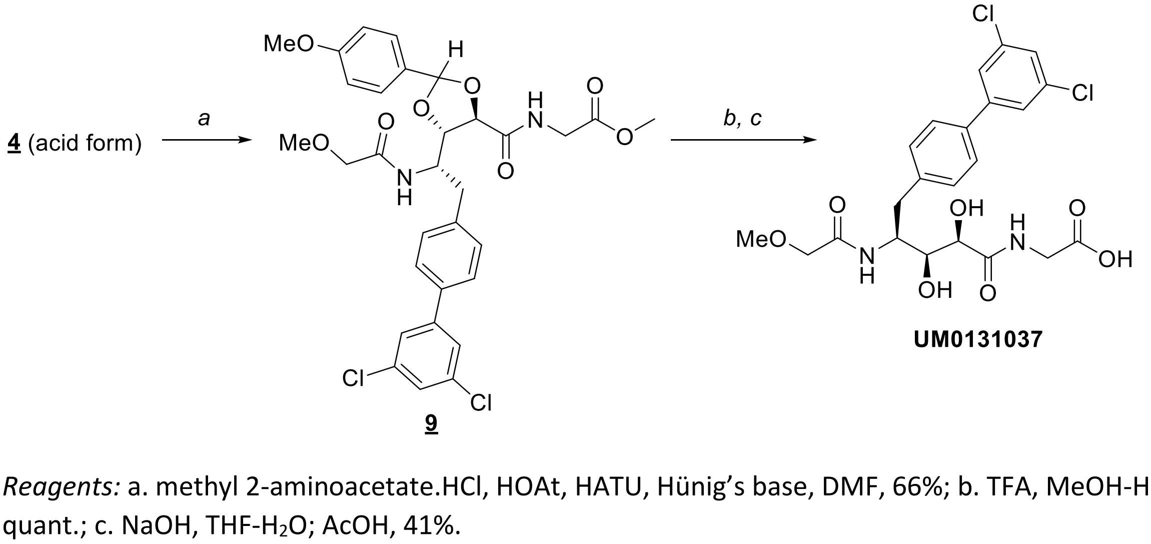

Reagents: a. methyl 2-aminoacetate.HCl, HOAt, HATU, Hünig's base, DMF, 66%; b. TFA, MeOH-H_2O, quant.; c. NaOH, THF-H_2O; AcOH, 41%.

Supplementary Figure 10. UM0131037 synthesis: Amide coupling of the acid form of intermediate **4** with methyl 2-aminoacetate afforded the ester **9**. Acid-mediated deprotection of the acetal of **9**, followed by saponification of the ester, then afforded **UM0131035**.

Supplementary Table 2: Compound Characterization Data

Compound	Analytical data
UM0128210	^{1}H NMR (400 MHz, $CDCl_3$): δ (ppm) 7.41 (d, *J*= 7.8 Hz, 2H), 7.33 (d, *J*= 1.6 Hz, 2H), 7.25 (t, *J*= 1.6 Hz, 1H), 7.20 (s, 1H), 7.18 (s, *overlapping with* $CHCl_3$ *peak*, 1H), 5.73 (br s, 1H), 4.21 (br s, 1H), 3.98 (br s, 1H), 3.57 (br s, 1H), 3.16 (m, 1H), 2.61 (m, 1H). HRMS (ESI-TOF) *m/z*: $[M+H]^+$ Calcd for $C_{17}H_{16}Cl_2NO_3$ 352.0507; Found 352.0510.
UM0128608	^{1}H NMR (400 MHz, $CDCl_3$): δ (ppm) 7.63 (d, *J*= 8.2 Hz, 2H), 7.45 (m, 6H), 7.40 (m, 2H), 7.36 (d, *J*= 7.8 Hz, 1H), 7.27 (m, 1H), 5.21 (br s, 1H), 4.17 (s, 2H). HRMS (ESI-TOF) *m/z*: $[M+H]^+$ Calcd for $C_{20}H_{17}Cl_2N_2O$ 371.0718; Found 371.0740.
UM0128610	^{1}H NMR (400 MHz, $CDCl_3$): δ (ppm) 8.16 (s, 1H), 7.45 (d, *J*= 8.2 Hz, 2H), 7.39 (m, 4H), 7.26 (s, 1H), 4.11 (q, *J*= 7.4 Hz, 2H), 3.98 (s, 2H), 3.25 (s, 2H), 1.20 (t, *J*= 7.4 Hz). HRMS (ESI-TOF) *m/z*: $[M+H]^+$ Calcd for $C_{18}H_{19}Cl_2N_2O_3$ 381.0773; Found 381.0794.
UM0131031	^{1}H NMR (400 MHz, $CDCl_3$): δ (ppm) 7.49 (br s, 1H), 7.44-7.34 (m, 4H), 7.29-7.23 (m, 3H), 7.18 (br s, 1H), 7.05 (br s, 1H), 4.58 (m, 1H), 4.45 (m, 1H), 4.22 (m, 1H), 4.18-3.99 (m, 1H), 3.96-3.80 (m, 1H), 3.79-3.66 (m, 2H), 3.41-3.30 (m, 1H), 2.87 (m, 1H), 1.48 (d, *J*= 7.0 Hz, 3H), 1.43 (t, *J*= 3.5 Hz, 3H). HRMS (ESI-TOF) *m/z*: $[M+H]^+$ Calcd for $C_{26}H_{32}Cl_2N_3O_8$ 584.1566; Found 584.1579.
UM0131035	^{1}H NMR (400 MHz, MeOH-d_4): δ (ppm) 7.55-7.50 (m, 4H), 7.39-7.34 (m, 3H), 4.37 (m, 1H), 4.16 (dd, *J*= 12.1, 1.2 Hz, 1H), 4.06 (dd, *J*= 18.0, 12.5 Hz, 1H), 4.02 (d, *J*= 5.9 Hz, 1H), 3.96 (d, *J*= 3.1 Hz, 1H), 3.94-3.88 (m, 2H), 3.82 (d, *J*= 15.3 Hz, 1H), 3.70 (d, *J*= 15.3 Hz, 1H), 3.34-3.26 (m, *overlapping with MeOH peak*, 1H), 3.29 (s, 3H), 2.84 (m, 1H). HRMS (ESI-TOF) *m/z*: $[M+H]^+$ Calcd for $C_{24}H_{28}Cl_2N_3O_8$ 556.1253; Found 556.1450.
UM0131037	^{1}H NMR (400 MHz, MeOH-d_4): δ (ppm) 7.90 (d, *J*= 9.0 Hz, 1H), 7.55 (d, *J*= 1.6 Hz, 2H), 7.53 (d, *J*= 8.2 Hz, 2H), 7.38 (s, 1H), 7.36 (d, *J*= 8.2 Hz, 2H), 4.35 (m, 1H), 4.14 (s, 1H), 4.07 (d, *J*= 18.0 Hz, 1H), 3.91 (d, *J*= 18.0 Hz, 1H), 3.90 (d, *J*= 9.0 Hz, 1H), 3.82 (d, *J*= 15.3 Hz, 1H), 3.70 (d, *J*= 15.3 Hz, 1H), 3.29 (s, 3H), 2.84 (dd, *J*= 13.7, 9.8 Hz, 1H). HRMS (ESI-TOF) *m/z*: $[M+H]^+$ Calcd for $C_{22}H_{25}Cl_2N_2O_7$ 499.1039; Found 499.1139.
